# A Rare but Reversible Cause of Hematemesis: “Downhill” Esophageal Varices

**DOI:** 10.1155/2016/2370109

**Published:** 2016-02-18

**Authors:** Lam-Phuong Nguyen, Narin Sriratanaviriyakul, Christian Sandrock

**Affiliations:** ^1^Division of Pulmonary, Critical Care, and Sleep Medicine, University of California, Davis, Suite #3400, 4150 V Street, Sacramento, CA 95817, USA; ^2^Department of Internal Medicine, University of California, Davis, Sacramento, USA; ^3^VA Northern California Health Care System, Mather, USA

## Abstract

“Downhill” varices are a rare cause of acute upper gastrointestinal bleeding and are generally due to obstruction of the superior vena cava (SVC). Often these cases of “downhill” varices are missed diagnoses as portal hypertension but fail to improve with medical treatment to reduce portal pressure. We report a similar case where recurrent variceal bleeding was initially diagnosed as portal hypertension but later found to have SVC thrombosis presenting with recurrent hematemesis. A 39-year-old female with history of end-stage renal disease presented with recurrent hematemesis. Esophagogastroduodenoscopy (EGD) revealed multiple varices. Banding and sclerotherapy were performed. Extensive evaluation did not show overt portal hypertension or cirrhosis. Due to ongoing bleeding requiring resuscitation, she underwent internal jugular (IJ) and SVC venogram in preparation for transjugular intrahepatic portosystemic shunt (TIPS), which demonstrated complete IJ and SVC occlusion. She underwent balloon angioplasty with stent placement across SVC occlusion with complete resolution of her varices and resolved hematemesis. “Downhill” varices are extremely rare, though previously well described. Frequently, patients are misdiagnosed with underlying liver disease. High index of suspicion and investigation of alternative causes of varices is prudent in those without underlying liver diseases. Prompt diagnosis and appropriate intervention can significantly improve morbidity and mortality.

## 1. Introduction

Esophageal varices can be associated with conditions other than liver disease and portal hypertension. There are three different types of esophageal varices, classified based on direction of venous flow: “uphill,” “downhill,” or idiopathic. The most common type, “uphill,” esophageal varices are caused by portal vein hypertension with subsequent collateral, decompressive flow. “Downhill” varices are rare dilated veins resulting from obstruction of the superior vena cava (SVC) leading to redirected blood flow to collateral system [1]. “Downhill” esophageal varices account for 0.4–11% of esophageal varices but less than 0.1% of patients present with hematemesis [2–4]. Often these cases of “downhill” variceal bleed are initially misdiagnosed and thought to be secondary to portal hypertension and/or liver disease. We report a case where recurrent variceal bleeding was initially misdiagnosed with portal hypertension but later found to have SVC thrombosis.

## 2. Case Presentation

**Figure 1:**
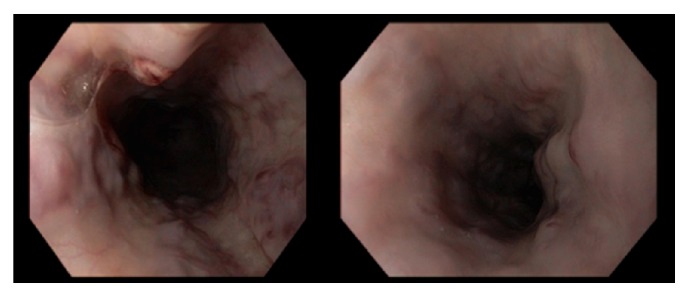
Initial EGD with extensive esophageal varices from the mid to distal esophagus.

A 39-year-old African American female with history of diabetes, hypertension, end-stage renal disease (ESRD) on hemodialysis (HD), and recurrent AV fistula thrombosis presented to our institution with recurrent hematemesis. Of note, she was started on dialysis in May of 2012, initially through a tunnel dialysis catheter approximately for one year until her AV fistula matured. She was recently discharged from the intensive care unit one week before for hematemesis and hematochezia. Esophagogastroduodenoscopy (EGD) during previous hospitalization showed 4 columns of large esophageal varices 25 cm to the distal esophagus just above the GE junction. There were nipple sign and red wale signs appreciated on the proximal portion of the varices suggesting recent bleeding for which banding and sclerotherapy were performed (Figure 1) with excellent hemostasis. The patient vomited up one cup of bright red blood but denied any associated dizziness, lightheadedness, abdominal pain, or diarrhea. She had been in her usual state of health since recent hospital discharge and denied any history of NSAIDs use, alcohol intake, or smoking history.

Upon presentation, she was afebrile with temperature 37.5°C, blood pressure 150/80 mmHg, heart rate 102–115/min, respiratory rate 16–21/min, and pulsed oximetry 91–100% on room air. Her physical exam was notable for an obese, chronically ill appearing female in no apparent distress. Head and neck exam was notable for facial and upper chest fullness with associated bilateral upper extremities swelling present for several months though the exact duration was unclear to the patient. Cardiac exam revealed evidence of tachycardia, distant heart sounds but no evidence of murmurs. Pulmonary exam was notable for diminished breath sounds at both bases but otherwise without crackles or wheezes. The remainder of her physical exam was unremarkable and without stigmata of liver disease such as telangiectasia, spider nevi, or palmar erythema.

Laboratory studies revealed leukocytosis with a white blood cell count of 18.1 × 10^9^/L with 85% neutrophils, 7% lymphocytes, and 7.5% monocytes as well as hemoglobin of 7.4 g/dL (baseline 11.8 g/dL) and platelets of 127 × 10^9^/L and no atypical lymphocytes. Her blood chemistry test results were notable for blood urea nitrogen of 42 mmol/L, serum creatinine 5.84 mg/dL, albumin 24 g/L, alkaline phosphatase, aspartate aminotransaminase, and alanine aminotransferase which were all within normal limits. Her INR was 1.1 on admission.

**Figure 2:**
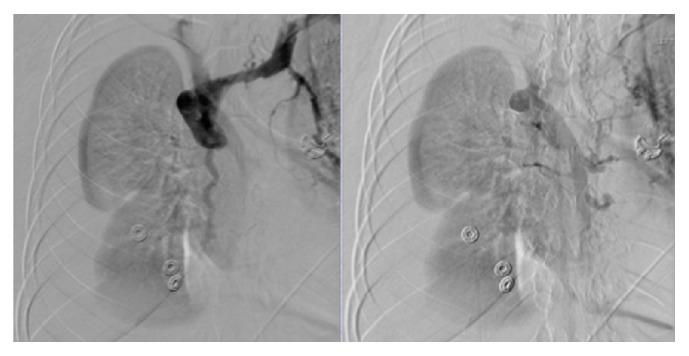
SVC venogram with occluded SVC and extensive collateral vessels.

**Figure 3:**
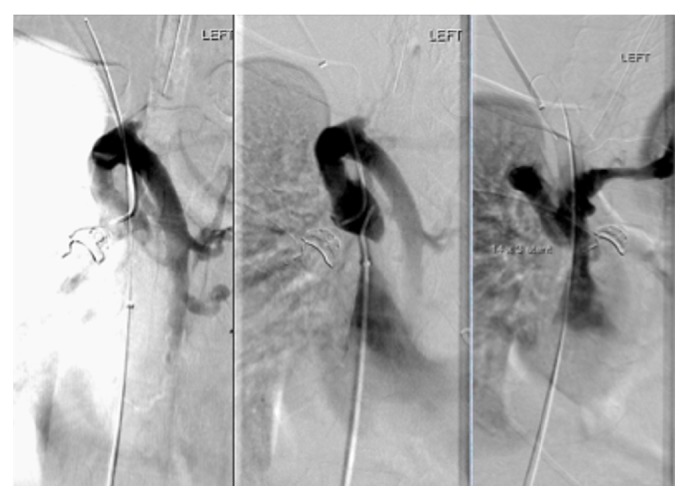
SVC venogram showing recannulization and stenting of distal occluded SVC.

**Figure 4:**
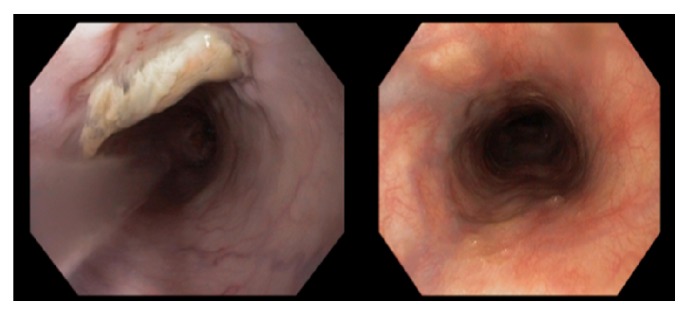
One month and four months after IR recannulization and stenting SVC with resolution of varices.

She was admitted to the intensive care unit for treatment and further evaluation for recurrent hematemesis of unclear etiology but presumed recurrent variceal bleeding. The patient underwent extensive evaluation including abdominal ultrasound with doppler showing patent main portal veins but evidence to suggest left portal vein portal hypertension. However, her liver CT showed normal hepatic anatomy with patent portal and hepatic veins, though there were extensive varices in the abdominal wall and mesentery. Due to continuous bleeding, requiring ongoing resuscitation and blood transfusion, a transjugular intrahepatic portosystemic shunt (TIPS) was planned. Internal jugular (IJ) and SVC venogram in preparation for TIPS were performed, which found complete occlusion of her right IJ and SVC (Figure 2). She was subsequently diagnosed with “downhill” esophageal varices due to SVC syndrome and underwent balloon angioplasty with 3 cm × 14 mm Smart stent placement across SVC occlusion (Figure 3) with resolution of her bleeding. Subsequent EGDs later showed complete resolution of her varices (Figure 4).

## 3. Discussion

“Downhill” esophageal varices account for 0.4–11% of esophageal varices and are commonly due to obstruction of the SVC as a result of direct compression or thrombosis [5]. Bleeding from downhill varices can be extremely rare and there have been less than 20 reported cases in literature. They either are located in the upper esophagus or may involve the entire esophagus depending on the level of SVC obstruction. If obstruction is proximal to the azygos vein, drainage can occur through mediastinal collaterals which resulted in varices limited to the upper portion of the esophagus [3]. If distal to the azygos vein, venous drainage occurs via the esophageal plexus leading to varices along the entire esophagus.

There are many reported etiologies of SVC obstruction. Most cases of SVC obstruction are associated with some forms of malignancy including lung, lymphoma, and mediastinal metastases [6, 7]. Nonmalignancy etiologies include pacemaker implantation [8], goiter [9, 10], central venous catheters (dialysis catheter in particular) [11–14], rheumatic heart disease [15], congenital heart disease [16], thymoma [5], and mediastinal fibrosis [17]. There are however no direct association or reported cases linking hypercoagulable state as a contributing cause.

Currently there are no definitive recommendations on screening and management of “downhill” varices. Hemostasis from variceal bleeding is often achieved with endoscopic local intervention such as banding and sclerotherapy [1, 2, 18]. The principle and definitive treatment is to relieve obstruction and revascularize SVC. Percutaneous radiological SVC angioplasty with stent placement had been reported with some success [19]. Underlying primary etiologies of SVC occlusion will need to be addressed and optimized. Finally surgical approach may be indicated for those with tumor causing extrinsic compression such as those with underlying thymoma or goiter.

Exact etiology of our patient's SVC thrombosis remains unclear despite extensive workup. However, she previously had a tunnel central venous dialysis catheter for nearly a year but it was removed two years ago prior to her current presentation. It is possible that her prior TDC catheter could have contributed to the development of her SVC thrombus. Incidences of central venous stenosis and obstruction secondary to dialysis catheter have been reported to be as high as 30% [20, 21]; however, SVC obstruction leading to hematemesis is exceedingly rare and usually occurs after the catheter has been in place for well over 2 years unlike our patient. Our patient's SVC thrombosis could have been present for quite some time prior to her current presentation as the patient was experiencing several months of chest, neck, upper extremities, and facial swelling indicating poor venous return. Her workup included chest imaging which revealed no central or mediastinal masses and serological workups including autoimmune and lupus anticoagulant were all negative. Decision was made to perform angioplasty and stent placement in this case to urgently relieve the SVC obstruction and improve her symptoms, especially in the setting of ongoing hematemesis. Anticoagulation was not initiated after stent placement due to several reasons. First she had recent life-threatening bleeding. Second, there were no hypercoagulable diseases identified. Lastly she was non-complaint to follow-up and periodically misses her dialysis. Her esophageal varices resolved on subsequent EGD four weeks later. Management of these patients with SVC thrombosis and obstruction can be extremely challenging as there are no current treatment guidelines and it is unclear when or if the stent can be safely removed. We present this case to raise clinician awareness of the alternative causes of varices and the challenges we face in management of these patients. High index of suspicion and investigation of alternative causes of varices is prudent in those without underlying liver diseases, as prompt diagnosis and appropriate intervention can significantly improve outcome.

## Abbreviations

EGD: Esophagogastroduodenoscopy
HD: Hemodialysis
IJ: Internal jugular
NSAIDs: Nonsteroidal anti-inflammatory drugs
SVC: Superior vena cava
TIPS: Transjugular intrahepatic portosystemic shunt.
